# The effects of GLP-1 analogues in obese, insulin-using type 2 diabetes in relation to eating behaviour

**DOI:** 10.1007/s11096-015-0219-8

**Published:** 2015-11-23

**Authors:** Stefanie Amarens de Boer, Joop Daniel Lefrandt, Japke Frida Petersen, Hendrikus Hessel Boersma, Douwe Johannes Mulder, Klaas Hoogenberg

**Affiliations:** 1 Department of Internal Medicine, Martini Hospital, Groningen, Netherlands; 2 Department of Vascular Medicine, University Medical Center Groningen, University of Groningen, Groningen, Netherlands; 3 Department of Clinical Pharmacy and Pharmacology, University Medical Center Groningen, University of Groningen, Groningen, Netherlands

**Keywords:** Diabetes mellitus, Eating behaviour, GLP-1 receptor agonist, Insulin therapy, Weight loss

## Abstract

*Background* Glucagon-like peptide-1 receptor agonists (GLP-1 RA) added to insulin in type 2 diabetes patients have shown to lower body weight, improve glycaemic control and reduce total daily insulin dose in short term studies, although the individual response greatly varies. *Objective* To evaluate GLP-1 RA treatment on body weight, glycaemic control and total daily insulin dose in obese, insulin-using type 2 diabetes patients after 2 years follow-up in a real life setting and to explore a possible relation with eating behaviour. *Setting* The Martini Hospital and the University Medical Center in Groningen in the Netherlands. *Methods* Eligible patients were at least 18 years of age, were on insulin therapy and obese (BMI > 30 kg/m^2^), started GLP-1 RA treatment. At baseline eating behaviour was classified according to the validated Dutch Eating Behaviour Questionnaire. A 2 years follow-up was performed. *Main outcome measures* Body weight, HbA1c and total daily insulin dose. *Results* 151 Patients started with exenatide or liraglutide. 120 patients completed the 2 years follow-up. From baseline to 2 years, body weight (mean ± SD) changed from 117.9 ± 22.1 to 107.9 ± 22.9 kg (*P* < 0.0001), HbA1c (median, IQR) changed from 7.9 (7.2–8.9) to 7.6 (6.9–8.3) % [63 (55–74) to 60 (52–67) mmol/mol] (*P* < 0.0001), total daily insulin dose changed from 90 (56–150) to 60 (0–100) Units/day (*P* < 0.0001). Weight change differed between eating behaviour groups (*P* < 0.001) in which external eating behaviour (*n* = 17) resulted in the smallest decline (−3.1 %) and restrained (n = 41) in the greatest (−10.3 %) in comparison with emotional (*n* = 37, −8.5 %) and indifferent (*n* = 25, −9.6 %) eating behaviours. *Conclusion* Two year of GLP-1 RA treatment resulted in a sustained reduction of weight, HbA1c and total daily insulin dose in obese, insulin-using type 2 diabetes patients in a real life setting. Largest weight loss was achieved in patients with a predominant restraint eating pattern while a predominant external eating pattern resulted in the smallest weight reduction.

**Electronic supplementary material:**

The online version of this article (doi:10.1007/s11096-015-0219-8) contains supplementary material, which is available to authorized users.

## Impacts on practice

- Addition of glucagon-like peptide-1 receptor agonists (GLP-1 RA) to obese, insulin-treated type 2 diabetes patients markedly reduces body weight, HbA1c and daily insulin doses or insulin discontinuation in a long term clinically based setting.
- Preexistent eating behavior modifies the amount of weight loss and assessment of eating behavior may help to identify those patients who will benefit most from GLP-1 RA.

## Introduction

Glucagon-like peptide-1 receptor agonists (GLP-1 RA) are used for weight loss and insulin dose reduction in obese insulin-using type 2 diabetic patients [1–7]. However, in daily practice the individual response of weight loss to GLP-1 RA varies greatly [8]. A recent Dutch cohort study [1] confirmed two earlier clinical based observational studies [2, 3] of a more than expected weight reduction than generally reported in randomized controlled trials [4–7]. A high BMI and longer diabetes duration at start of treatment has been identified as predictors of greater weight loss [8] but other factors involved are unknown.

A plausible mechanism by which GLP-1 RA may induce weight loss is by suppressing appetite signalling in the brain and increasing satiety, leading to a reduced food intake [9, 10]. GLP-1 receptors are present in the central nervous system suggesting direct actions of GLP-1 in the brain [11]. GLP-1 infusions can enhance satiety and reduce energy intake in type 2 diabetes patients [12]. Furthermore, GLP-1 RA attenuates binge eating in obese patients [13], suggesting a role of GLP-1 RA in certain eating types.

The psychology of eating distinguishes three main types of eating behaviours i.e. predominant external, emotional, and restrained eating patterns. External eaters are triggered in response to sensory stimuli irrespective of satiety [14]. Emotional eaters are driven by stress and emotions while the natural response would be to loose appetite [15]. Restrained eaters intentionally limit food intake to control weight. However, the self-imposed food restriction in restrained eaters is recognised by the body as true food shortage which goes into the starvation mode thus increasing hunger and lowering metabolic rate [16]. All these three eating behaviour patterns have been implicated in the risk of developing obesity [17]. A possible relation between eating patterns and response to GLP-1 RA has never been studied in patients with type 2 diabetes on insulin.

## Aim of the study

To explore the role of eating behaviour on changes in weight, glycaemia and total daily insulin dose (TDD) in obese, insulin-using type 2 diabetes patients in clinical practise.

## Ethical approval

The study was notified by the local ethics committee and was performed in accordance with the principles of the Declaration of Helsinki. All patients consented with the protocol including regular outpatient follow-up (FU) and the off-label use of GLP-1 RA with insulin.

## Methods

### Study design and participants

This prospective observational cohort study recruited patients at the Martini Hospital Groningen and the University Medical Center Groningen in the Netherlands. Eligible subjects were obese (BMI > 30 kg/m^2^), insulin-using type 2 diabetes with a long-standing wish of weight reduction. They were >18 years of age, had a diabetes duration for >1 year and were receiving long-, intermediate or short-acting insulin (insulin glargine, insulin detemir, NPH insulin, regular insulin, insulin aspart, insulin lispro, or mixed insulin) with or without oral antidiabetic drugs (OAD). Patients were excluded if they had recurrent hypoglycaemia, a history of bariatric surgery, a history of pancreatitis, or had been treated with anti-obesity medication. The goals of GLP-1 RA treatment were weight loss and insulin dose reduction. The first patient started GLP-1 RA in January 2008 and the last patient started in January 2011. The study sample size was reflective of the number of eligible patients during this period.

### Study protocol

GLP-1 RA treatment was initially given with exenatide, dose increased to 10 µg twice daily, which was at the start of the study in January 2008 the only available GLP-1 RA in the Netherlands. In May 2009, liraglutide was the second GLP-1 RA marketed in the Netherlands and preferred by patients because of its once daily administration, dose increased to 1.8 mg per day. At that time it was decided to study both GLP-1 RA with the intention to make a between GLP-1 RA drug comparison on outcomes.

No specific dietary restrictions were given for the study other than that all patients had received previous instructions and education on a healthy diet and lifestyle in accordance with the Dutch standards of care. FU was done at 6 weeks, 3, 6, 9, 12, 18 months, and after 2 years. At all study visits HbA1c (%), body weight (kg) and blood pressure were measured (mmHg), adherence to GLP-1 RA therapy, TDD (U/day), and OAD were documented. Adverse events were also noted including hypoglycaemic events. Hypoglycaemia was defined as major if the patient needed assistance for treatment of hypoglycaemia. At the start and after 2 years of FU serum creatinine (umol/l), triglycerides (mmol/l), HDL-, LDL-, and total cholesterol (mmol/l) were measured.

### Dutch Eating Behaviour Questionnaire

At baseline, eating behaviour was classified according to the validated Dutch Eating Behaviour Questionnaire (DEBQ) [18]. The DEBQ has a scale on restrained eating (e.g. “Do you try to eat less at mealtimes than you would like to eat?”) and two separate scales on overeating tendency: emotional eating (e.g. “Do you have a desire to eat when you are irritated?”) and external eating (e.g. “If food smells and looks good, do you eat more than usual?”) [18]. Patients were classified according to their predominant eating pattern as defined by van Strien [18] as restrained, emotional, external or indifferent eaters.

### Outcome measures

The primary outcome variable was change in weight after 2 years FU. Secondary outcome measures were change in HbA1c and TDD after 2 years FU. Safety parameters included: hypoglycaemia, nausea, vomiting, diarrhoea, and hospital admission.

### Statistical analysis

Data are presented as numbers and percentage. Variables with a normal distribution are presented as mean ± SD and otherwise as median and inter-quartile range (IQR). Statistical analyses were performed using the Statistical Package for Social Sciences version 20 (SPSS Inc., Chicago, Iln, USA). Variables at baseline and after 2 years of treatment were compared using the Students paired *t* test and a Wilcoxon test were required. To compare groups, the Fisher exact or a one way ANOVA was used (or a Kurskall Wallis test if necessary), with logarithmic transformation for non-normally distributed variables and a post hoc Bonferroni for multiple comparisons if significant. To compare multivariate means between groups, a MANOVA was used. Backward multiple linear stepwise regression was used to identify variables independently (*P* < 0.100) associated with change in weight, HbA1c, and TDD. Logistic regression was used to identify variables independently associated with stopping insulin treatment. Since the duration of diabetes and BMI may be potential confounders, subgroup analyses were performed by dividing the study cohort into tertiles of these variables. A *P* < 0.05 was considered statistically significant.

## Results

One hundred and fifty-one obese, insulin-using type 2 diabetes patients were started on GLP-1 RA therapy. One hundred and twenty patients completed the 2 years of FU, 21 stopped ≤6 months mainly due to side-effects (mostly gastrointestinal) and because they experienced no effects on weight and insulin dose. Nine patients were lost to FU, and one patient died due to myocardial infarction. The present analysis was done in those patients (n = 120) that completed 2 year FU after the initial prescription.

### Clinical characteristics

Table 1 shows the clinical characteristics at baseline and after 2 years FU. Mean age was 58.4 ± 8.1 years, 52.5 % were females, and the median diabetes duration was 10.0 (7.0–16.0) years. From baseline to 2 years, body weight (mean ± SD) changed from 117.9 ± 22.1 to 107.9 ± 22.9 kg (*P* < 0.0001), HbA1c (median, IQR) changed from 7.9 (7.2–8.9) to 7.6 (6.9–8.3) mmol/l [63 (55–74) to 60 (52–67) mmol/mol] (*P* < 0.0001), TDD changed from 90 (56–150) to 60 (0–100) Units/day (*P* < 0.0001) and 30 % (*n* = 36) of the patients were able to stop insulin treatment. A total of 57 patients (47.5 %) experienced side effects, mostly gastrointestinal including nausea and vomiting (*n* = 45), diarrhoea (*n* = 3), constipation (*n* = 1). Two patients had to be hospitalized because of acute renal failure following severe dehydration due to a lack of intake. No severe hypoglycaemia occurred.

**Table 1:** Clinical characteristics at baseline and change after 2 years

Characteristics	At baseline	After 2 years	*P* value^a^
N	120		
Female (n)	63 (52.5 %)		
Age (years)	58.4 (8.1)		
Diabetes duration (years)	10 (7.0–16.0)		
BMI (kg/m^2^)	39.5 (6.5)	36.1 (6.2)	<0.0001
Weight (kg)	117.9 (22.1)	107.9 (21.9)	<0.0001
HbA1c (%)	7.9 (7.2–8.9)	7.6 (6.9–8.3)	<0.0001
HbA1c (mmol/l)	63 (55–74)	60 (52–67)	
Total cholesterol (mmol/l)	4.0 (3.6–4.6)	3.8 (3.4–4.6)	0.011
HDL (mmol/l)	1.1 (0.9–1.3)	1.1 (0.9–1.3)	0.834
LDL (mmol/l)	2.1 (1.6–2.5)	2.1 (1.6–2.5)	0.601
Triglycerides (mmol/l)	1.94 (1.47–3.08)	1.77 (1.31–2.35)	<0.0001
Serum creatinine (umol/l)	74.5 (66.0–88.8)	74.0 (62.0–85.0)	0.006
Systolic blood pressure (mmHg)	140.0 (130–148)	133.0 (120–140)	<0.0001
Diastolic blood pressure (mmHg)	80.0 (75.0–88.0)	80.0 (70.0–80.0)	<0.0001
Diabetes treatment			
Insulin therapy (n)	120 (100 %)	84 (69.4 %)	<0.0001
Insulin dose (U/day) (all patients, n = 120)	90 (56–150)	60.0 (0–100)	<0.0001
Insulin dose (U/kg/day) (all patients, n = 120)	0.75 (0.47–1.33)	0.54 (0.00–0.87)	<0.0001
Insulin dose (U/day) (patients using insulin after 2 years, n = 84)	119 (73–182)	80 (55–124)	<0.0001
Insulin dose (U/kg/day) (patients using insulin after 2 years, n = 84)	1.01 (0.65–1.47)	0.71 (0.53–1.11)	<0.0001
Oral therapy			
No oral therapy (n)	13 (10.8 %)	16 (13.3 %)	0.453
1 oral therapy (n)	80 (66.7 %)	86 (71.7 %)	0.307
2 oral therapy (n)	27 (22.5 %)	18 (15 %)	0.049
GLP-1 treatment			
Exenatide (n)	73 (60.8 %)	56 (46.7 %)	
Liraglutide (n)	47 (39.2 %)	64 (53.3 %)	

Data are expressed as number (%) or means (SD) or if not normal distributed as median IQR

^a^ *P* < 0.05 indicates statistical significance

### Exenatide and liraglutide treatment

Of the 120 patients completing the 2 years FU, 73 patients started with exenatide and 56 patients started with liraglutide. A total of 18 patients switched from exenatide to liraglutide when liraglutide became available and one patient switched from liraglutide to exenatide. Subgroup analysis revealed no differences in clinical characteristics of patients using exenatide or liraglutide, according to treatment at baseline and after 2 years FU (Supplemental table S1). Exenatide and liraglutide showed similar weight losses [exenatide (mean, SD) −11.6 ± 8.3 kg vs liraglutide −8.8 ± 7.5 kg, *P* = 0.058], similar reductions in HbA1c (exenatide −0.54 ± 1.4 % vs liraglutide −0.43 ± 1.0 %, *P* = 0.641), similar reductions in insulin dose [exenatide (median, IQR) −31 (−60 to −7) U/day vs liraglutide −45 (−63 to −15) U/day, *P* = 0.119] all according to the treatment received at 2 years FU. Since weight loss, HbA1c and insulin dose reduction were similar, the two GLP-1 treatments, exenatide and liraglutide, were combined in the further analysis.

### Change in weight

**Fig. 1:**
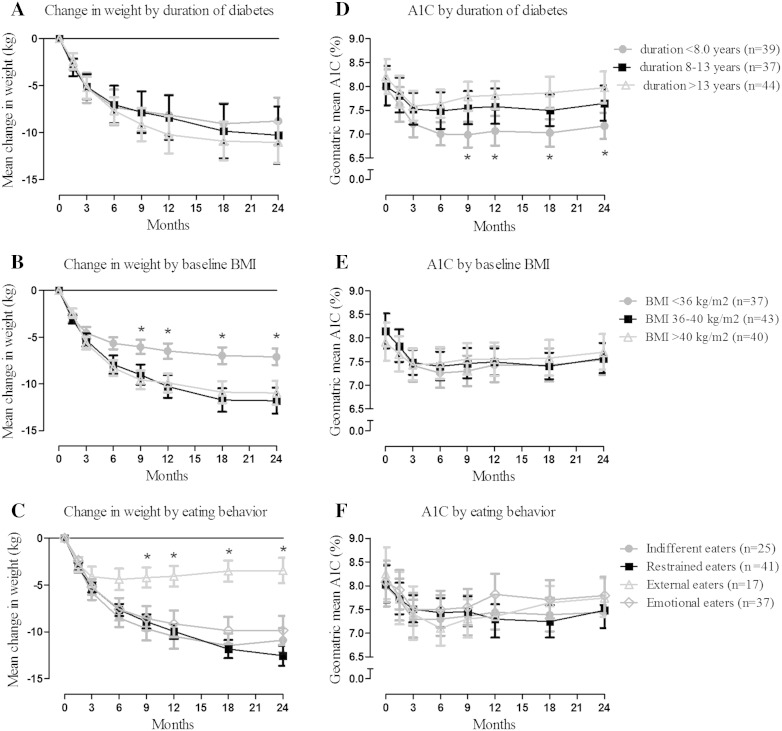
Change in weight (kg) according to duration of diabetes (**a**), baseline BMI (**b**) and eating behaviour group (**c**); HbA1c (%) according to duration of diabetes (**d**), baseline BMI (**e**) and eating behaviour group (**f**) in the patients (n = 120) who completed the 2 years follow-up. *Data* are presented as mean change in weight (SEM) and geometric mean (95 % CI). *Asterisk* denotes *P* < 0.05 to *P* < 0.01 from other groups

The overall changes in weight according to different predefined groups (diabetes duration, BMI and eating behaviour) are given in Fig. 1a–c. There were no significant differences in weight changes among diabetes duration tertiles. Across baseline BMI tertiles, a greater weight loss was observed in patients with a higher baseline BMI (36–40 and >40 kg/m^2^) which was significant at 9 months (*P* = 0.03), 12 months (*P* = 0.025), 18 months (*P* = 0.010) and 2 years FU (*P* = 0.021). According to eating behaviour, the smallest decline was observed in external eaters which was significant at 9 months (*P* = 0.032), 12 months (*P* = 0.010), 18 months (*P* = 0.001), and 2 years FU (*P* = 0.001).

Multiple linear stepwise regression analysis was used to identify the predictors of the change in weight at 2 years FU. Only baseline BMI, beta (95 % CI) −0.230 (−0.399 to −0.062, *P* = 0.008) was significantly associated with weight loss. Sex, age, baseline HbA1c, baseline TDD, and diabetes duration were not significant and were excluded from the model.

### Glycaemic control

The overall change in HbA1c (geometric mean and 95 % CI) according to the predefined subgroups are given in Fig. 1d–f. Across diabetes duration tertiles, a lower HbA1c was observed in patients with a shorter diabetes duration (<8 years) which was significant at 9 months (*P* = 0.001), 12 months (*P* = 0.003), 18 months (*P* = 0.001), and 2 years FU (*P* = 0.002). There were no significant differences in HbA1c among baseline BMI tertiles and eating behaviour groups.

Multiple linear stepwise regression analysis was used to identify the factors (eating behaviour excluded) associated with change in HbA1c at 2 years FU. Only baseline HbA1c beta (95 % CI) −0.587 (−0.733 to −0.441, *P* < 0.0001), and diabetes duration beta 0.191 (0.043–0.338, *P* = 0.011) were independently associated with changes of HbA1c. Sex, age, baseline BMI, and baseline TDD were not significant and were excluded from the model. Moreover, there was no relation between change in weight (kg) and change in HbA1c (%) at 2 years FU, Pearson correlation 0.013, *P* = 0.887.

### Insulin dose

The decrease in TDD throughout the 2 years of FU (Table 1) across the diabetes duration tertiles, was lower in patients with a shorter diabetes duration (<8 years) (*P* < 0.0001 at every point of FU). Across baseline BMI tertiles, patients with a lower BMI (36 < kg/m^2^) used significantly (*P* < 0.05) less Units/day at every point of FU. The change in TDD did not differ between baseline BMI tertiles and eating behaviour groups.

Multiple linear stepwise regression analysis was used to identify predictors (except for eating behaviour) of the change in TDD at 2 years FU. Only baseline TDD, beta (95 % CI) −0.450 (−0.279 to −0.622, *P* < 0.0001) and diabetes duration, beta 0.176 (0.005–0.347, *P* = 0.044) were significant associated with changes of TDD, if tested stepwise. Sex, age, baseline HbA1c, and baseline BMI were not significant and were excluded from the model. In the logistic regression analysis, only diabetes duration (years) with an odds-ratio (95 % CI) of 0.893 (0.814–0.979, *P* = 0.016) and TDD (Units) with an odds-ratio of 0.976 (0.964–0.988, *P* < 0.0001) were associated with stopping insulin treatment, whereas sex, age, baseline HbA1c, and baseline BMI were not independently associated.

### Eating behaviour

**Fig. 2:**
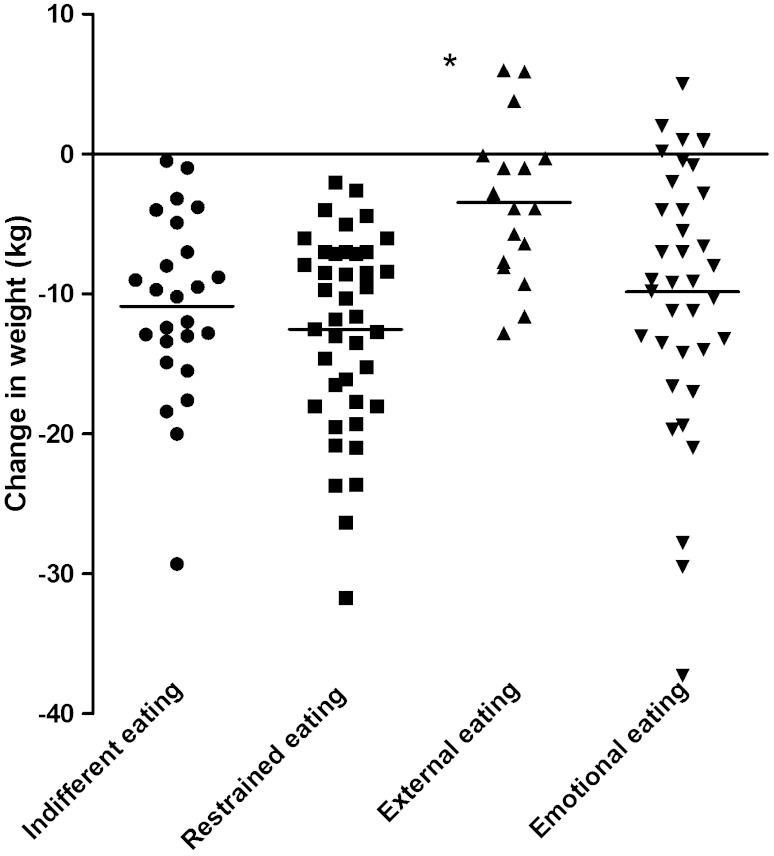
Individual changes in weight at 2 years of follow-up according to eating behaviour category. *Asterisk* denotes *P* < 0.05 from other groups

In each group of eating behaviour, body weight was significantly reduced after 2 years FU: external (*n* = 17) 117.8 ± 18.7 kg to 114.4 ± 20.4 kg (−3.1 %, *P* < 0.022), emotional (*n* = 37) 113.8 ± 19.7 kg to 103.9 ± 19.0 kg (−8.5 %, *P* < 0.001), restrained (*n* = 41) 123.8 ± 22.7 kg to 111.3 ± 22.5 kg (−10.3 %, *P* < 0.001), indifferent (*n* = 25) 114.6 ± 25.4 kg to 103.8 ± 24.8 kg (−9.6 %, *P* < 0.001). Weight change at 2 years FU differed significantly between types of eating behaviour (Fig. 2, *P* < 0.001). After correction for differences between different types of eating behaviour (Supplemental table S1) in baseline BMI, diabetes duration, baseline HbA1c, and baseline TDD, the differences in change in weight at 2 years FU remained significant (MANOVA, *P* < 0.001). Changes in HbA1c and TDD at 2 years FU did not differ significantly between types of eating behaviour. If tested with a one way ANOVA and post hoc Bonferroni predominant external eaters showed less change in weight which was significant at 9 months (*P* = 0.032), 12 months (*P* = 0.010), 18 months (*P* = 0.010) and 2 years FU (*P* = 0.010) compared to emotional, restrained and indifferent predominant eaters.

## Discussion

The current study demonstrated that GLP-1 RA given to obese, insulin-using type 2 diabetes patients resulted in a marked weight loss, improved glycaemic control and considerably reduced daily insulin doses. These changes were most prominent during the first 3 months of GLP-1 RA treatment, then gradually tapered and sustained during 2 years of follow-up. Thereby these findings confirm and expand other clinical based cohort studies [1–3] that showed a more than average weight loss on GLP-1 RA than generally reported [4–7]. In addition, we found that pre-existing eating behaviour influenced GLP1 RA induced weight loss, which was obvious in restrained and indifferent eaters and mitigated in patients with an external eating trait.

GLP-1 RA have consistently shown to reduce weight and improve glycaemic control in insulin-using type 2 diabetes patients, however variable responses have been reported [4–7]. Two systematic reviews mentioned a −0.9 to −5.6 kg and a −1.5 to −4.9 kg (−3.2 kg on average) weight loss when GLP-1 RA was added to insulin in type 2 diabetes patients [6, 7]. Our study demonstrated a much larger weight loss that corresponded with the amount of weight reduction of −6.5 (±0.8) kg at 26 weeks [2], −12.8 (±7.5) kg [3] and −14.3 (±9.5) kg [1] at 52 weeks at the consecutive time points in our study.

The GLP-1 RA therapy was given either with exenatide, an exendin-based GLP-1 receptor agonist, or with liraglutide, a human GLP-1 analogue, for reasons connected to the clinical based observational nature of our study (see “Methods” section). A comparison of the efficacy of both treatment options disclosed no meaningful differences in outcome of our study. This is in conjunction with the RCT that head-to-head compared exenatide and liraglutide (LEAD-6 trial) [19] showing similar weight loss of both drugs. The LEAD-6 trial found larger HbA1c reductions with liraglutide than with exenatide but in a quite different type 2 diabetic patient population only using OAD treatment and no insulin.

Little is known about the factors that determine the amount of weight loss by GLP-1 RA treatment. In a post hoc analysis on a previous RCT, the greatest reductions in weight and also HbA1c were seen in the more obese patients (BMI > 30 kg/m^2^) and the longest diabetes duration (>13 years) [8]. In conjunction, we found the greatest weight reductions in the most obese patients (BMI > 36 kg/m^2^) and a non-significant tendency for a longer diabetes duration (>13 years). Thus the substantial weight loss that we and the few other studies observed, may have resulted from higher baseline BMI that amply exceeded 35 kg/m^2^ in all [1–3]. A confounding factor may have been the selection of patients as we included those in whom overweight complicated their diabetes with weight reduction as the primary treatment goal. Therefore a selection bias may have occurred as some patients who did not experience an early clinical benefit from GLP-1 RA discontinued the study. Since our study was observational in nature with no control group, it is difficult to determine the exact weight loss attributable to the GLP-1 RA treatment. Notwithstanding that the currently observed weight loss was that what genuinely can be achieved in outpatient clinical setting, as recently reported [1].

To our knowledge, this is one of the first studies that investigated eating behaviour in relation to efficacy of GLP-1 RA administration showing that restrained and indifferent eaters obviously lost weight and external eaters had the smallest reductions in weight loss. There is only one small Japanese study in obese type 2 diabetes patients (n = 16) that assessed eating scores and traits in relation to the GLP-1 RA, showing that amount of weight loss correlated with a promptly reduced score for the sense of hunger that showed an interaction with external eating behaviour [20].

There are a few studies that might provide a pathophysiological basis by which external eating mitigated weight reduction. First, in an functional magnetic resonance imaging (fMRI) study healthy obese men were treated with exenatide or saline infusion in a blinded fashion and shown food pictures (external eating stimulus). Only in those subjects who showed ≥ 10 % reduction in caloric intake (responders), effects of exenatide were observed on the fMRI in the hypothalamus [21]. Second, in another fMRI study the brain responses to food pictures were higher in obese non-diabetic and type 2 diabetes than lean subjects [10]. GLP-1 RA administration decreased the brain responses to food pictures and food intake in the obese non-diabetic and type 2 diabetes subjects and a lower reduction in food picture-induced brain activation (external stimulus) led to a smaller decrease in food intake [10]. In terms of external eating behaviour this means that persistent brain activation was marginally diminished by GLP-1 RA and did not change food intake. Hence from these experimental studies, we suppose that a higher proportion of external eaters could have diminished or lacking effects of GLP1-RA on the brain.

The DEBQ is a widely used questionnaire that was developed to measure the three eating behaviour styles which are generally accepted as the psychological basis of overeating. It has been shown that patients with obesity, anorexia nervosa, and healthy controls all have a different eating behaviour as measured by the DEBQ [22]. Interestingly, reduced GLP-1 levels in bulimia nervosa have been associated with binge-eating episodes [23]. As binge-eating is common in restrained and in emotional eaters, this may give an indication why these eating behaviour patterns in our study benefited from GLP-1 RA treatment.

The diabetes treatment was individually tailored by the treating physicians (JDL, KH, KMT, SHJD). Despite the limitation that no strict treatment protocol for glycaemic control was used, net glycaemia improved, TDD decreased with an average of 30 U/day with 30 % of patients stopping insulin and no severe episodes of hypoglycaemia. Thus this clinical based study provided an impression of the expected benefit on HbA1c and insulin dose reduction that can be achieved by GLP1 RA treatment in daily practise.

## Conclusion

This observational cohort study showed that GLP-1 RA treatment resulted in a sustained reduction of weight, HbA1c and TDD in obese insulin-using type 2 diabetes patients in a real life setting. Largest weight loss was achieved in patients with a predominant restraint eating pattern while a predominant external eating pattern resulted in the smallest weight reduction. The finding that pre-existing eating behaviour modulated the effects of GLP1-RA on weight loss deserves further study.

## Electronic supplementary material

Below is the link to the electronic supplementary material.

Supplementary material 1 (DOCX 19 kb)
