# Supplementary material for: The effects of GLP-1 analogues in obese, insulin-using type 2 diabetes in relation to eating behaviour
Source: Int J Clin Pharm. 2015 Nov 23;38(1):144–51. doi: 10.1007/s11096-015-0219-8 (PMC4733138; doi:10.1007/s11096-015-0219-8)
Supplement: Supplementary file 1 — Supplementary material 1 (DOCX 19 kb) [file 11096_2015_219_MOESM1_ESM.docx]

**Supplemental table S1. Clinical characteristics of the study subjects according to treatment**

| **Characteristics** | | **Exenatide** | **Liraglutide** | **P value^a^** |
| --- | --- | --- | --- | --- |
| **N** | **Baseline** | 73 (60.8%) | 56 (46.7%) |  |
|  | **2 year FU** | 47 (39.2%) | 64 (53.3%) |  |
| **Female (n)** | **Baseline** | 35 (47.9%) | 28 (59.6%) | 0.262 |
|  | **2 year FU** | 26 (46.6%) | 37 (57.8%) | 0.272 |
| **Age (years)^b^** | **Baseline** | 58.4 (8.4) | 58.0 (7.9) | 0.996 |
|  | **2 year FU** | 58.0 (8.3) | 58.7 (8.0) | 0.657 |
| **Diabetes duration (years)^c^** | **Baseline** | 10.0 (7-14) | 11.0 (6.0-18) | 0.537 |
|  | **2 year FU** | 9.5 (7-14) | 11.0 (7.0-16.8) | 0.382 |
| **BMI (kg/m^2^)** | **Baseline** | 39.2 (6.2) | 37.5 (7.0) | 0.528 |
|  | **2 year FU** | 35.2 (6.1) | 36.9 (6.3) | 0.148 |
| **Weight (kg)** | **Baseline** | 115.7 (22.0) | 116.3 (22.3) | 0.506 |
|  | **2 year FU** | 107.4 (21.6) | 108.3 (22.3) | 0.828 |
| **HbA_1c_ (%)** | **Baseline** | 7.8 (7.1-8.6) | 8.3 (7.4-9.0) | 0.215 |
| **HbA_1c_ (mmol/l))** |  | 62 (54-70) | 67 (57-75) |  |
|  | **2 year FU** | 7.6 (6.7-8.1) | 7.6 (7.0-8.3) | 0.227 |
|  |  | 60 (50-65) | 60 (53-67) |  |
| **Insulin dose (U/day)** | **Baseline** | 83 (56-143) | 108 (52 -155) | 0.368 |
|  | **2 year FU** | 60 (0.0-90) | 61 (0.0-100) | 0.996 |

Data are expressed as number (%) or means (SD) or if not normal distributed as median IQR.
^a^*P* < 0.05 indicates statistical significance. ^b^ Age (years) at start treatment. ^c^ Diabetes duration at start treatment.
